# The antioxidant effect of triptolide contributes to the therapy in a collagen-induced arthritis rat model

**DOI:** 10.1080/13510002.2021.2004047

**Published:** 2021-11-17

**Authors:** Guang-Min Yu, Li-Feng Zhou, Bi-Xia Zeng, Jing-Jun Huang, Xiao-jun She

**Affiliations:** a School of Biomedical and Pharmaceutical Sciences, Guangdong University of Technology, Guangzhou, People’s Republic of China; b Department of Gastroenterology, Second Clinical Hospital of North Sichuan Medical College, Nanchong Central Hospital, Nanchong, People’s Republic of China; c School of Chemical Engineering and Light Industry, Guangdong University of Technology, Guangzhou, People’s Republic of China

**Keywords:** Triptolide, rheumatoid arthritis, rat, collagen induced arthritis, local oxidative stress, systemic oxidative stress, bone destruction, relative weight of organ

## Abstract

**Background:**

As a chronic autoimmune disease, rheumatoid arthritis (RA) is related to oxidative stress, which may lead to the occurrence and persistence of inflammation in RA. The purpose of this study is to evaluate the potential antioxidant effect of triptolide in collagen-induced arthritis (CIA) rat model.

**Methods:**

We examined the severity of arthritis, levels of local and systemic oxidative stress, periarticular bone erosion and weight of organs in CIA rats treated with triptolide.

**Results:**

We found that triptolide decreased the paw thickness and clinical arthritis score, significantly. The mRNA expression and activity of myeloperoxidase and inducible nitric oxide synthase were remarkably decreased in the paws of the CIA rats after triptolide treatment. Triptolide significantly inhibited the levels of nitrite and nitrate in serum, as well as the urinary level of dityrosine. Triptolide treatment also markedly increased bone volume of tibia, but suppressed epiphyseal plate thickness of both femur and tibia. In addition, there was no significant difference in the weight of organs after the therapy, except decreased spleen weight.

**Conclusions:**

These results suggested that the local and systemic oxidative stress was enhanced in the CIA rats and the therapeutic dose of triptolide had a definite antioxidant effect.

## Introduction

As early as the seventeenth century, people had a preliminary understanding of this disease, but it was not until 1859 that Dr. Alfred Garrod named it rheumatoid arthritis (RA) [1]. RA is a chronic and systemic autoimmune disease, which leads to severe inflammation and destruction of the joint architecture [2]. It affects approximately 1% of the world’s population, mainly women [3]. RA decreases the quality of life and work ability, causes considerable number of disabilities, and even raises the mortality rate [4]. Non-steroidal anti-inflammatory drugs, disease-modifying anti-rheumatic drugs, steroid and biological response modifiers are currently used for treating RA. However, it is not possible to achieve a satisfactory improvement in a certain group of patients and these drugs often bring serious side effects [5]. Therefore, searching for more effective and safer drugs is still a top priority for doctors and researchers.

**Figure 1.**
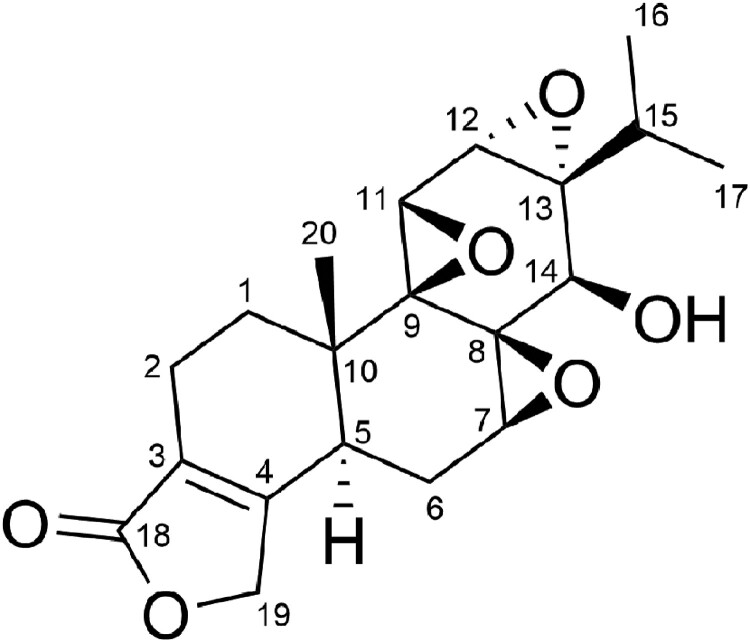
Chemical structure of triptolide.

Triptolide, a diterpene triepoxide in chemical structure (Figure 1), is extracted from the plant *Tripterygium wilfordii* Hook F [6]. Increasing experimental evidence has confirmed its anti-RA effect [7]. Its regulation of inflammation [8,9], cell proliferation [10,11], angiogenesis [12,13], and bone homeostasis [14,15,16] have been well studied, even its toxicity [17,18]. Recently, oxidative stress in RA has attracted great attention in the research community [19,20]. In this study, we focused on the antioxidant effect of triptolide during the therapy process. We examined whether triptolide has antioxidant effect on collagen-induced arthritis (CIA), which is a commonly used experimental model of RA [21]. Here, we demonstrate the effects of triptolide on the severity of arthritis, levels of local and systemic oxidative stress and periarticular bone erosion in the CIA rats. We also show the effect of triptolide on the organs weight of the CIA rats.

## Materials and methods

### Animals

Specific Pathogen Free-grade, female Wistar rats (8 weeks old) were purchased from Southern Medical University (Guangzhou, China). They were fed with standard laboratory chow and water *ad libitum* and kept under controlled conditions of temperature (24 ± 1°C), relative humidity (40–80%), and light (16 h light: 8 h dark cycle). All animal protocols were approved by the Institutional Animal Care and Use Committee of Guangdong University of Technology and followed the National Institutes of Health Guide for the Care and Use of Laboratory Animals.

### Collagen-induced arthritis (CIA) model and treatment

To establish a CIA model, bovine type II collagen (Chondrex Inc., Redmond, WA, USA) was dissolved in 0.05 M acetic acid overnight at 4°C, and emulsified on ice with an equal amount of incomplete Freund’s adjuvant (Chondrex Inc.). On day 0, the emulsion mixture (200 μg collagen/rat) was injected subcutaneously at the base of their tails, and then a second injection was performed on day 7, according to the manufacturer’s recommendations. The control group was injected with equal volumes of sterilized saline.

All rats were monitored for arthritis severity by body weight, paw thickness and clinical arthritis score. Each paw of the animal was scored from 0 to 4 as the following scoring system. ‘Score 0 = no erythema or swelling; score 1 = slight erythema or swelling of one of the toes or fingers; score 2 = erythema and swelling of more than one toe or finger; score 3 = erythema and swelling of the ankle or wrist; score 4 = complete erythema and swelling of toes or fingers and ankle or wrist [22].’ A mean score was given to each animal. On day 21, only the established rats (with clinical scores of 3–4) were randomly assigned to receive sterilized saline or triptolide by gavage (n = 6 for each group).

From day 22, the rats were given 45 μg/kg/d triptolide (Sanling Biotech., Guilin, China) dissolving in sterilized DMSO (Sigma, St. Louis, MO, USA) by gavage for 28 days [13]. On day 50, rats were euthanatized under anesthesia with intraperitoneal administration of pentobarbital sodium (Huayehuanyu Chem., Beijing, China). Lives, spleens, thymuses and adrenals were then harvested and weighed immediately.

### Quantitative polymerase chain reaction (qPCR)

Total RNA was extracted from joint tissue using TRIzol reagent (Life Technologies, Grand Island, NY, USA) and reverse transcribed using M-MLV 1st Stand Kit (Invitrogen, Waltham, MA, USA) [23,24]. qPCR was performed using SYBR Green Mix (Life Technologies) on a LightCycler 96 Real-Time PCR System (La Roche, Basel, Switzerland) according to the method described previously [25]. Normalization was performed using the housekeeping gene β-actin as a control. The primer sequences are listed in Table 1.

**Table 1.** Primers used for qPCR.

Genes	Accession numbers	Primer sequence (5′–3′)
*β-actin*	XM_039089807.1	Forward: ACCACCATGTACCCAGGCATT
		Reverse: CCACACAGAGTACTTGCGCTCA
*MPO*	NM_001107036.1	Forward: ACCTACCCCAGTACCGATCC
		Reverse: AACTCTCCAGCTGGCAAAAA
*COX-2*	AF233596.1	Forward: TGTATGCTACCATCTGGCTTCGG
		Reverse: GTTTGGAACAGTCGCTCGTCATC
*iNOS*	AY211532.1	Forward: CACCACCCTCCTTGTTCAAC
		Reverse: CAATCCACAACTCGCTCCAA

### Colorimetric assay

The activities of myeloperoxidase (MPO), cyclooxygenase-2 (COX-2), and inducible nitric oxide synthase (iNOS) in the supernatant of joint tissue lysate were determined by commercial kits (Abcam, Cambridge, UK) as per the manufacturer’s instructions.

The levels of nitrite and nitrate in serum were measured by a colorimetric assay kit (Dojindo, Tokyo, Japan). They were determined by spectrophotometric analysis at 540 nm (TriStar^2^ S LB 942 Multimode Reader; Berthold Technologies, Bad Wildbad, Germany) with reference to a standard curve [26].

### Enzyme-linked immunosorbent assay (ELISA)

Sample of urine was collected from each rat approximately 30 min before euthanasia and centrifuged at 12,000 rpm for 10 min at 4°C. The urine sample was stored at −80°C until analysis. The level of dityrosine in urine was analyzed with an ELISA kit (JaICA, Shizuoka, Japan). To normalize dityrosine, the other kit (Oxford Biomedical Research, Oxford, MI, USA) was used to detect the creatinine level in the urine sample.

### Image of microfocal computed tomography (μCT)

After euthanasia, the hind limb was removed and fixed with 4% paraformaldehyde for 24 h. The sample was scanned by μCT (MILabs, Utrecht, Netherlands). Histomorphometric analysis was conducted on distal femur and proximal tibial. Bone volume fraction (bone volume/total volume, BV/TV), subchondral bone plate thickness and epiphyseal plate thickness were measured by a software (IMALYTICS Preclinical 2.1, Utrecht, Netherlands).

### Statistics analysis

Statistical analyses were carried out by the Student’s *t*-test and one-way analysis of variance (ANOVA), followed post hoc Tukey’s test for multiple groups comparison (GraphPad Prism 5; GraphPad Software Inc., La Jolla, CA, USA), because all the variables were normally distributed. Data are expressed as mean ± standard deviation (S.D.). A value of *P* < 0.05 was considered statistically significant.

## Results

### The anti-CIA effect of triptolide in rats

**Figure 2.**
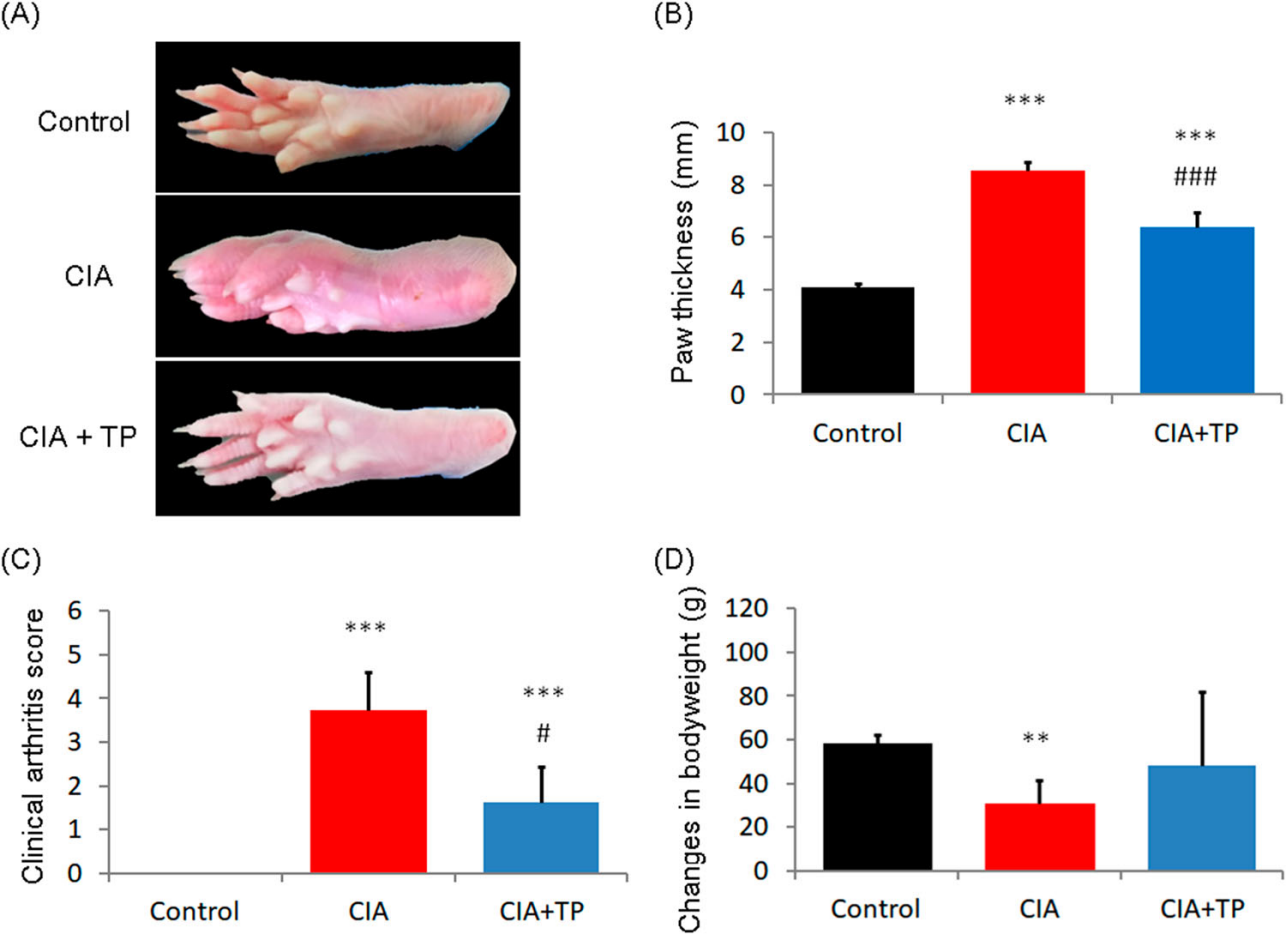
Effect of triptolide on the severity of arthritis in CIA rats. (a) Representative photos of the control rats and the CIA rats treated with sterilized saline and triptolide; (b) paw thickness; (c) clinical arthritis score; and (d) changes in body weight of the rats at the end of drug administration. CIA: collagen-induced arthritis rats treated with sterilized saline. CIA + TP: collagen-induced arthritis rats treated with triptolide. Data are expressed as mean ± S.D. (*n* = 6). **P < 0.01 and ***P < 0.001 compared with the control group. ^#^*P *< 0.05 and ^###^*P *< 0.001 compared with the CIA group.

The paw morphology of different groups at the end of the experiment is shown in Figure 2(a). Figures 2(b,c) show after 28 days of treatment, triptolide exhibited a significant anti-CIA effect according to the paw thickness (*P *< 0.001) and clinical arthritis score (*P *< 0.05). As shown in Figure 2(d), the weight of CIA rats changed seriously (*P *< 0.01). However, the use of triptolide prevented weight lost.

### Triptolide inhibits the level of local oxidative stress

**Figure 3.**
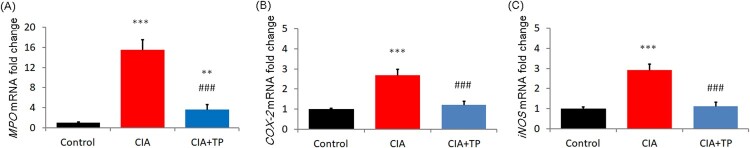
Effect of triptolide on the mRNA expression levels of *MPO*, *COX-2*, and *iNOS* in the articular tissue. CIA: collagen-induced arthritis rats treated with sterilized saline. CIA + TP: collagen-induced arthritis rats treated with triptolide. Data are expressed as mean ± S.D. (*n* = 6). **P < 0.01 and ***P < 0.001 compared with the control group. ^###^*P *< 0.001 compared with the CIA group.

To investigate the effect of triptolide on local oxidative stress, the levels of MPO, COX-2, and iNOS in articular tissue were evaluated. The mRNA expression of *MPO*, *COX-2* and *iNOS* increased markedly (*P *< 0.001) in the CIA rats; triptolide treatment significantly reduced (*P *< 0.001) their levels (Figure 3). Similarly, after administration of triptolide, the activities of MPO and iNOS were also substantially suppressed (*P *< 0.001) in the joint tissue (Table 2).

**Table 2.** Effect of triptolide on the levels of MPO, COX-2, and iNOS in the articular tissue.

Iterms	Control	CIA	CIA + TP
MPO (pg/mg tissue)	17.70 ± 2.12	124.08 ± 8.85***	37.52 ± 3.01***^,^^###^
COX-2 (ng/mg tissue)	20.00 ± 6.15	63.08 ± 15.38**	35.38 ± 7.69
iNOS (ng/mg tissue)	13.04 ± 2.37	48.01 ± 2.96***	21.34 ± 2.96*^,^^###^

CIA: collagen-induced arthritis rats treated with sterilized saline; CIA ± TP: collagen-induced arthritis rats treated with triptolide.

Data are expressed as mean ± S.D. (*n* = 6).

*P < 0.05, **P < 0.01, and ***P < 0.001 compared with the control group. ^###^*P *< 0.001 compared with the CIA group.

### Triptolide decreases the level of systemic oxidative stress

The effect of triptolide on the level of systemic oxidative stress in the CIA rats was also studied. As shown in Table 3, serum levels of nitrite and nitrate in the CIA rats were clearly higher (*P *< 0.001) than those in the control rats. However, after treatment with triptolide, their levels decreased significantly (*P *< 0.05). In addition, the level of dityrosine increased substantially (*P *< 0.001) in urine of the CIA rats, but was markedly reduced (*P *< 0.001) by triptolide treatment that followed.

**Table 3.** Effect of triptolide on the level of oxidative stress in serum and urine.

Iterms	Control	CIA	CIA + TP
Nitrite + nitrate (μM)	14.24 ± 1.32	21.57 ± 1.75***	16.19 ± 1.75^#^
Dityrosine (pmol/mg Cr)	41.31 ± 1.99	58.18 ± 3.42***	31.29 ± 3.59**^,^^###^

CIA: collagen-induced arthritis rats treated with sterilized saline; CIA ± TP: collagen-induced arthritis rats treated with triptolide.

Data are expressed as mean ± S.D. (*n* = 6).

**P < 0.01 and ***P < 0.001 compared with the control group. ^#^*P *< 0.05 and ^###^*P *< 0.001 compared with the CIA group.

### Triptolide reduces bone destruction

**Figure 4.**
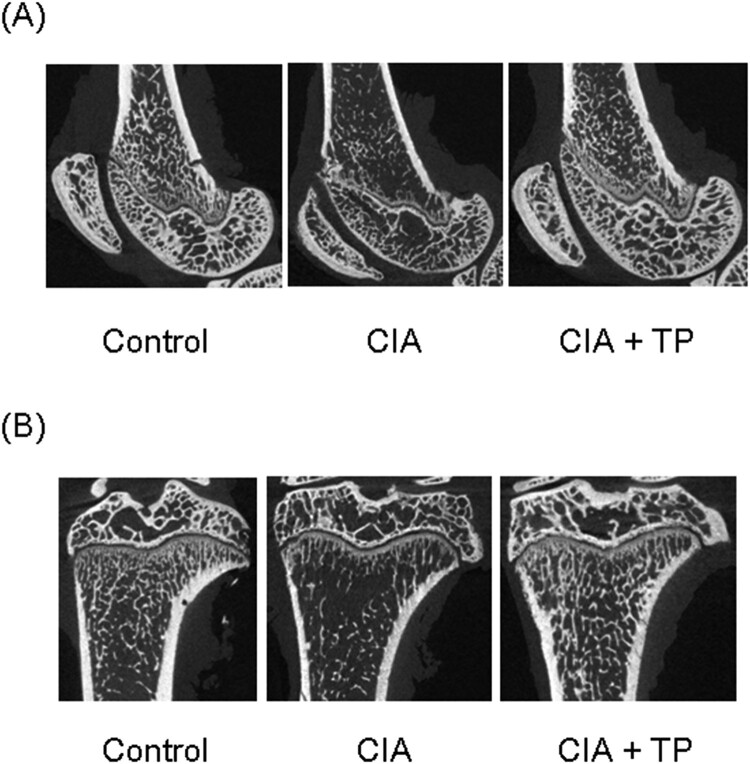
Effect of triptolide on periarticular bone erosion in knee joints. Images show bones of (A) femur and (B) tibia. CIA: collagen-induced arthritis rats treated with sterilized saline. CIA + TP: collagen-induced arthritis rats treated with triptolide.

The effect of triptolide on bone structure was verified by μCT scan. Representative images are shown in Figure 4. The CIA rats exhibit severe damage of articulation. However, triptolide significantly suppressed its degree. BV/TV of femur and tibia in the CIA rats was substantially lower (*P *< 0.05 and *P *< 0.01, respectively) than that in the control rats (Table 4). The use of triptolide significantly increased (*P *< 0.05) BV/TV of the tibia. There was no statistical difference in the thickness of subchondral bone plate of femur. However, the tibial subchondral bone plate was significantly thinner (*P *< 0.01) in the CIA rats. The epiphyseal plates of femur and tibia were significantly thicker (*P *< 0.01 and *P *< 0.05, respectively) in the CIA rats; triptolide treatment significantly decreased (*P *< 0.01 and *P *< 0.05, respectively) their thickness.

**Table 4.** Effect of triptolide on BV/TV, subchondral bone plate thickness and epiphyseal plate thickness of distal femur and proximal tibia.

Bones	Iterms	Control	CIA	CIA + TP
Femur	BV/TV (%)	25.9 ± 3.2	17.1 ± 3.7*	24.9 ± 5.1
	Subchondral bone plate (μm)	120.9 ± 54.7	100.5 ± 35.5	118.6 ± 33.3
	Epiphyseal plate (μm)	194.2 ± 11.1	262.5 ± 26.6**	204.4 ± 9.2^##^
Tibia	BV/TV (%)	26.5 ± 3.7	14.0 ± 2.5**	22.5 ± 3.1^#^
	Subchondral boneplate (μm)	137.9 ± 19.2	59.3 ± 18.8**	89.9 ± 27.7
	Epiphyseal plate (μm)	178.9 ± 8.29	228.9 ± 27.0*	175.6 ± 15.0^#^

CIA: collagen-induced arthritis rats treated with sterilized saline; CIA ± TP: collagen-induced arthritis rats treated with triptolide.

Data are expressed as mean ± S.D. (*n* = 6).

*P < 0.05 and **P < 0.01 compared with the control group. ^#^*P *< 0.05 and ^##^*P *< 0.01 compared with the CIA group.

### Triptolide modulates the weight of organs in CIA rats

As shown in Table 5, the relative weight of liver in the rats with triptolide treatment was significantly heavier (*P *< 0.05) than that in the CIA rats. There was no significant difference between the control rats and the CIA rats or the control rats and the rats treated with triptolide. No statistical difference in the relative weight of spleen was detected between the control rats and the CIA rats. However, it was significantly lower (*P *< 0.05) in the rats after triptolide treatment than in the control rats and the CIA rats. In addition, the relative weight of thymus and adrenal in the CIA rats were markedly higher than those in the control rats (*P *< 0.05). There was no statistical difference between the control rats and the rats treated with triptolide.

**Table 5.** Effect of triptolide on the weight of organs in CIA rats.

Iterms	Control	CIA	CIA + TP
Relative weight of liver (%)	3.57 ± 0.07	3.40 ± 0.08	3.59 ± 0.07^#^
Relative weight of spleen (%)	0.232 ± 0.004	0.236 ± 0.006	0.216 ± 0.005*^,^^#^
Relative weight of thymus (%)	0.215 ± 0.014	0.183 ± 0.007*	0.200 ± 0.007
Relative weight of adrenal (%)	0.0322 ± 0.0012	0.0361 ± 0.0014*	0.0336 ± 0.0014

Relative weight was the organ weight/body weight.

CIA: collagen-induced arthritis rats treated with sterilized saline; CIA ± TP: collagen-induced arthritis rats treated with triptolide.

Data are expressed as mean ± S.D. (*n* = 6).

*P < 0.05 compared with the control group. ^#^*P *< 0.05 compared with the CIA group.

## Discussion

At present, there is no ideal method to treat RA, and the potential mechanism of the existing targeted drugs is unclear [27]. Accumulating researches have confirmed the anti-inflammatory effect of triptolide and scientifically explained its therapeutic effect on RA [8,9]. However, inflammatory responses activate the production of immune-modulators, leading to drastic changes in the level of oxidative stress [24]. Effective anti-inflammatory treatment strategies should be able to not only cut down the synthesis of inflammatory mediators but also the oxidative stress resulted by inflammation [28]. In the present study, we confirmed the antioxidant effect of triptolide on CIA rats during the therapy process.

In this study, we chose the articular tissue to assess local oxidative stress. MPO, a well-known enzyme, is synthesized by activated neutrophils and monocytes, stored in azurophilic granules and released into the phagosome or the extracellular fluid to catalyze the conversion of hydrogen peroxide to hypochlorous acid [29]. It characterized by strong pro-oxidative and pro-inflammatory properties [30]. Here, we report for the first time that triptolide inhibits *MPO* mRNA expression and activity in the CIA rats, which may have a beneficial effect on this disease. It is an ideal that an anti-inflammatory drug with selective inhibition of COX-2 activity [31]. Inducible COX-2 is the rate limiting enzyme in the synthesis of prostaglandin E_2_ (PGE_2_). Our results show triptolide decreased *COX-2* mRNA level in the CIA rats, and there was no significant difference in COX-2 activity between the rats treated with triptolide and the normal rats. Similarly, previous studies have shown that triptolide can suppress the production of PGE_2_ by selectively inhibiting the COX-2 level in the inflamed joints and serum of CIA rats [32,33]. The iNOS is the isoform of key enzyme that responsible for regulating the pathologic role of nitric oxide (NO) [34]. It is a pretty important work showing that triptolide could suppress NO production by reducing the gene transcription of iNOS [35]. Results of the present study also show both mRNA level and activity of iNOS decreased in the paws of the rats treated with triptolide.

In the present study, we selected serum and urine samples to evaluate systemic oxidative stress. NO is an important signaling molecul involved in regulating a variety of physiological and pathological processes [36]. It reacts easily with oxidizing agents in serum to generate nitrite and even nitrate [37,38]. We detected higher levels of them in the CIA model rats than those in the control group, as they were elevated under most inflammatory conditions [39,40]. For the first time, our data suggest that triptolide inhibited the development of CIA by holding back the formation of nitrite and nitrate. Dityrosine is an ideal marker of oxidatively modified proteins, as peroxynitrite can affect tyrosine dimerization and nitration [41,42]. Our results suggest that triptolide suppressed systemic oxidative protein damage in the CIA rats. To our knowledge, this is the first demonstration of the inhibitory effect of triptolide on dityrosine level in a RA animal model. Therefore, oral administration of triptolide can suppress CIA induced systemic oxidative stress.

Inflammation and oxidative stress are tightly connected. Under the condition of RA, there is a positive feedback between inflammation and oxidative stress, and the destructive effects of each other are amplified by these two participants. Nuclear factor-κB (NF-κB) not only increases the production of IL-1 and TNF-α, but can also be activated by these pro-inflammatory cytokines [19]. Furthermore, inflammatory responses cause drastic changes in the level of oxidative stress [24]. On the other hand, reactive oxygen species can also activate the NF-κB pathway; it is obvious that oxidative stress is related to the molecular signaling dysregulation in the early stage of RA [20]. However, the mechanism by which oxidative stress may lead to the occurrence and persistence of inflammation in RA remains to be investigated. And the role of antioxidants as RA therapies seems to be neglected because only a small number of researches have been found [19,20]. In the present study, triptolide treatment reduced the inflammatory processes in CIA rats, which may result in reduction of oxidative stress secondary.

Triptolide can not only efficiently resist oxidative stress but also prevent bone destruction, which has important clinical significance. Consistent with previous studies [13,14,43], we found that triptolide effectively reduced the severity of arthritis by mitigating the paw thickness and clinical arthritis score of the CIA rats. Data of μCT also validated that triptolide could decrease bone destruction in affected joints. Here, we first found the effect of triptolide on subchondral bone plate and epiphyseal plate. Results of the present study give further evidence that triptolide can protect both bone and cartilage of the inflammatory joints, effectively.

As the public is highly concerned about the safety of drugs [44], we also evaluated the effect of triptolide on the weight of organs in CIA rats, which is commonly used in toxicity tests. Contrast to some toxicological experiments, in which high doses of triptolide were used (0.2–600mg/kg) [45,46], we observed that there was no significant difference in the weight of organs after the therapy in this study, except for the decrease of spleen weight. Increased spleen weight could be seen in impaired spleens in the chronic toxicity test [47]. The nephrotoxicity induced by triptolide is also related to oxidative stress [17]. In sharp contrast to enhanced oxidative stress in toxicity tests, the therapeutic dose of triptolide has a definite antioxidant effect in the present study.

## Conclusions

The data of this study demonstrated that the local and systemic oxidative stress was enhanced in the CIA rats and the therapeutic dose of triptolide had antioxidant activity.

## Data Availability

All data analyzed during this study are available from the corresponding author on request.

## Abbreviations

**Abbreviations:** RA: rheumatoid arthritis; CIA: collagen-induced arthritis; qPCR: quantitative polymerase chain reaction. ELISA: enzyme-linked immunosorbent assay; MPO: myeloperoxidase; COX-2: cyclooxygenase-2; iNOS: inducible nitric oxide synthase; μCT: microfocal computed tomography; BV: bone volume; TV: total volume
